# When will we have enough evidence to require improvements at the workplace?

**DOI:** 10.5271/sjweh.4199

**Published:** 2024-12-01

**Authors:** Alex Burdorf

**Affiliations:** Department of Public Health, Erasmus MC Rotterdam, the Netherlands.

## The need for evidence-based improvements at the workplace

Work is an important social determinant of health (1). Decades of research on a wide range of different types of exposure, such as chemical, physical, biological, and psychosocial exposures at the workplace, has shown how poor working conditions are associated with adverse health outcomes and disparities in health. This has been well illustrated in the 2024 Discussion Paper series celebrating '50 years of research' in the *Scandinavian Journal of Work, Environment & Health* (2–9). With this rapidly increasing body of knowledge, it is quite disappointing to note that the increasing number of high-quality studies on identifying occupational risk factors are not matched by a similar number of high-quality studies aimed to design and evaluate measures that promote workers’ health and prevent work-related disease and disability (2).

There is a scarcity of evidence-based workplace interventions that can guide appropriate workplace policies, programs, and practices. A linked issue is that, in situations when sufficient evidence seems available, there is a lack of urgency in utilizing research findings in the practice of occupational safety and health (OSH). A European Union report argued that, in order for research to have an impact on workers’ safety and health, there is a great need to improve the translation of OSH research findings into practice, both in the occupational health services and at workplaces (10).

Both the lack of evidence-based guidance for healthy and safe workplaces as well as lack of implementation of available knowledge in practice can be partly attributed to the complexity of the workplace, given its multiple organizational levels, variety of settings and contextual factors, and required level of flexibility and adaptivity of interventions in order to be successful (11). Complex interventions require different research strategies, shifting the focus from the traditional binary question of effectiveness of a precisely defined intervention towards a broader understanding of mechanisms, processes, and outcomes of relevance to workers, companies, and OSH professionals (12).

## Rise and demise of the RCT

Many researchers have been trained that randomized controlled trials (RCT) provide the most rigorous evidence on beneficial effects of preventive, diagnostic, and therapeutic interventions (13). With the rise of the evidence-based medicine approach, the RCT design became the gold standard, demonstrating with high quality and reliability the causal effects of the intervention. In the past 40 years, this gold standard has increasingly been critiqued and – in the era of complex system thinking and complex interventions – the status of the RCT is eroding (13, 14). There is strong evidence that if evidence-based health care is limited to RCT, the evidence will favor individual-based over multicomponent interventions, highly standardized simple interventions over complex ones, and short- over long-term interventions (14).

In occupational health, it has long been acknowledged that interventions often have an organizational or work environment context that hampers – or even excludes the possibility of – individual-level randomization and also makes cluster randomization very difficult (15). Very successful interventions in occupational health, eg, the introduction of a complete ban on asbestos (3) and the substitution of organic solvents in paints (4), would not have happened if knowledge from an RCT would have been the required level of evidence.

## Alternative study designs and research methods

In recent years, there has been a big debate around which study designs are needed for a valid interpretation of an intervention's effects. At the heart of the discussion is the inherent trade-off between causal estimation of an intervention effect and estimation of an intervention effect that matters for practice. Opinions remain strongly divided.

Some researchers claim that observational studies will always be affected by bias and, thus, causal evidence can only be derived from experiments or trails emulated in observational studies (16). An experiment with randomization ensures exchangeability in that both groups are comparable for all known and unknown factors and, thus, these factors cannot bias the intervention effect. In the absence of an RCT, researchers have applied the ‘experimental causal inference’ framework to particular situations that may be interpreted as happening at random. These so-called 'natural experiments' have specific statistical methods that try to ensure that the intervention of interest can be interpreted as an exogenous source (17). These natural experiment approaches include, among others, methods such as propensity-score matching, difference-in-differences, interrupted time series, instrumental variables, and regression discontinuity. An illustrative example is the introduction of a new nationwide graded return-to-work program whereby propensity-score matching demonstrated an increasing labor force participation and reduced permanent disability as a result of this intervention (18). Laaksonen and colleagues (19) used the regression discontinuity approach to show that vocational rehabilitation, assigned based on a threshold in earnings in past few years, increased paid employment, but results were far from being statistically significant. A difference-in-differences approach comparing trends before and after the introduction of a work reintegration program demonstrated reductions in the number of disability days, most notably among longer duration episodes (20). Jan Vandenbroucke and colleagues (21) have eloquently criticized this ‘causal inference’ movement, stating that truly natural experiments are rare and those that happen are usually not widely generalizable. Hence, complete reliance on natural experiments and linked statistical methods will limit much needed evidence in occupational health tremendously (22).

On the other side of the spectrum, researchers argue for a paradigm shift towards more practice-relevant evidence with a system lens on multiple factors that interact in dynamic and unpredictable ways in the complex world of real data (23, 24). It is proposed to determine whether an intervention contributes, along with other factors, to a desirable outcome, acknowledging that multiple components of the intervention might each contribute to an overall beneficial effect through heterogeneous effects on disparate causal pathways (23). Such a question can often not be answered by a specific research design, but requires a myriad of new types of data, analytical methods, and interdisciplinary work (24). It will be of interest to see in the next few years whether the ‘experimentalists’ and the ‘system thinkers’ can bridge the huge gap in their appreciation of what is considered solid evidence.

## When is evidence strong enough to take action?

Societal impact of our research requires research that matters, ie, research that provides compelling arguments to implement specific improvements in order to contribute to the health and wealth of workers and communities. Working longer in good health is an example of a key societal challenges in many countries (25), and, thus, researchers in occupational health are in the fortunate position to create societal impact by identifying determinants of healthy working-life expectancy and designing interventions that promote working longer in good health. However, this requires that scientific scrutiny for valid results does not result in postponing highly needed programs and policies in the workplace.

Several articles celebrating '50 years of research' in the Journal have documented that some preventive measures have been delayed unnecessarily, sometimes in a deliberate strategy by industry to manufacture uncertainty (4, 26). Delays in taking necessary action can also be the result of scientists hesitating too long about the correct interpretation of results. A classic example is the infamous crocidolite-chrysotile asbestos debate during the 1980s and 1990s, whereby proponents of the 'chrysotile is safe' argument delayed the ban on asbestos (3). In an earlier editorial, we have argued that causal inference considerations should hamper neither development nor implementation of evidence-based recommendations in occupational health when the evidence-base is reasonable (27).

There are no golden rules to determine when our knowledge is sufficient to require preventive measures. I can only refer to the wise words of Sir Bradford Hill in his seminal address to the Royal Society of Medicine, describing the purpose of the newly-founded section of occupational medicine: “All scientific work is incomplete—whether it be observational or experimental. All scientific work is liable to be upset or modified by advancing knowledge. That does not confer upon us a freedom to ignore the knowledge we already have or to postpone action that it appears to demand at a given time (28)."

With these words in mind, we look forward to research that provides compelling information on how to tackle the current challenges in occupational health and studies that document how this knowledge has guided successfully preventive measures at the workplace. Hopefully, we can redress the imbalance between risk-oriented research and intervention studies and create a long-lasting positive societal impact on workers’ health.
